# Applying Deep Reinforcement Learning to Cable Driven Parallel Robots for Balancing Unstable Loads: A Ball Case Study

**DOI:** 10.3389/frobt.2020.611203

**Published:** 2021-02-22

**Authors:** Alex Grimshaw, John Oyekan

**Affiliations:** The Department of Automatic Control and Systems Engineering, The University of Sheffield, Sheffield, United Kingdom

**Keywords:** deep reinforcement learning, manufacture, pandemic, construction, cable robotics, q-learning, load, CDPR

## Abstract

The current pandemic has highlighted the need for rapid construction of structures to treat patients and ensure manufacturing of health care products such as vaccines. In order to achieve this, rapid transportation of construction materials from staging area to deposition is needed. In the future, this could be achieved through automated construction sites that make use of robots. Toward this, in this paper a cable driven parallel manipulator (CDPM) is designed and built to balance a highly unstable load, a ball plate system. The system consists of eight cables attached to the end effector plate that can be extended or retracted to actuate movement of the plate. The hardware for the system was designed and built utilizing modern manufacturing processes. A camera system was designed using image recognition to identify the ball pose on the plate. The hardware was used to inform the development of a control system consisting of a reinforcement-learning trained neural network controller that outputs the desired platform response. A nested PID controller for each motor attached to each cable was used to realize the desired response. For the neural network controller, three different model structures were compared to assess the impact of varying model complexity. It was seen that less complex structures resulted in a slower response that was less flexible and more complex structures output a high frequency oscillation of the actuation signal resulting in an unresponsive system. It was concluded that the system showed promise for future development with the potential to improve on the state of the art.

## Introduction

The current pandemic has highlighted the need for rapid construction of structures to treat patients and ensure manufacturing of health care products such as vaccines. To achieve this, currently, a large manpower is needed to achieve this. Nevertheless, this exposes the workers to the danger of catching a virus or acting as a carrier to future patients. In this work, we propose the use of a robotic platform called a cable driven parallel manipulator (CDPM) to rapidly build structures. Toward this, a control strategy is required to control the end effector of the robotic platform. Having been utilized since the 1950’s, reinforcement learning is one of the oldest fields of machine learning and artificial intelligence, yet in recent years it has been experiencing a resurgence as a framework for learning sequential decision tasks (Garychl, 2018). At the same time, cable driven parallel manipulators (CDPMs)—where flexible cables replace rigid links as robot actuators—are becoming increasingly popular for their numerous benefits (Saber, 2015). This project aims to introduce reinforcement learning into a CDPM to balance an object on a platform as it is moved from one location to another, with the hope of improving upon the state of the art. Specifically, a ball is to be balanced on a flat plate, controlled by eight cables spaced in pairs at equidistant intervals in a workspace that are driven by motors.

The developments of this project have the potential to improve the performance of cable balancing systems in areas such as warehouse swarm robot optimization, shipyard container movement management, drone auto-balancing and general robotic balancing (Gullapalli et al., 1994; NIST, 1994; Lachevre et al., 2017) by reducing operational times and failure rates. In this work, our contributions is as follows: We make use of reinforcement learning to enable the transport of a continuous moving load, a ball in this case, which could be highly unstable at large speeds during transport. This is important especially when CDPM are to be used in rapid construction of emergency structures.

## Background and Literature Review

### Cable Driven Parallel Manipulators

As defined by Gallardo-Alvarado (2016), a Parallel Manipulator (PM) is a mechanical system formed by two linked platforms, namely, the fixed platform and the moving platform. The moving platform is connected to the fixed platform by at least two independent computer-controlled serial chains or limbs working in parallel. Cable Driven Parallel Manipulators (CDPM) are a subsidiary of the standard parallel manipulator where rigid limbs are replaced with retractable cables allowing for varying limb length.

The properties of PMs and CDPMs provides unique advantages when applied in robotics. Patel and George (2012) discussed in their 2012 paper how parallel manipulators offer a greater load carrying capacity, low inertia, higher structural stiffness, and a reduced sensitivity to certain errors. Generally, parallel manipulators provide a clear advantage over most serial manipulation robots in that they control end effector position with a high degree of precision (Tannous et al., 2014), which makes them excellent for use in invasive surgical procedures where a high degree of precision is mandated (Beira et al., 2011). However, parallel manipulators have smaller and less dextrous workspaces due to link interference, where coupling of link actuation is resistive due to counteractive movement. When compared to PMs, CDPMs offer additional advantages due to the properties of the cables. These include a higher payload to weight ratio, larger workspace, higher end-effector speed and acceleration, and being easy to reconfigure and implement (Tang, 2014; Qian et al., 2018). Replacing rigid links with cables does introduce new challenges in their design, particularly in the precise control of the end-effector position (Tang, 2014; Qian et al., 2018), which becomes difficult to ensure due the need for cables to be constantly under tension (Bosscher and Ebert-Uphoff, 2004; Qian et al., 2018) and the elastic nature of cables. This problem can be partly mitigated through more complex controller design and specific material design choices.

**FIGURE 1:**
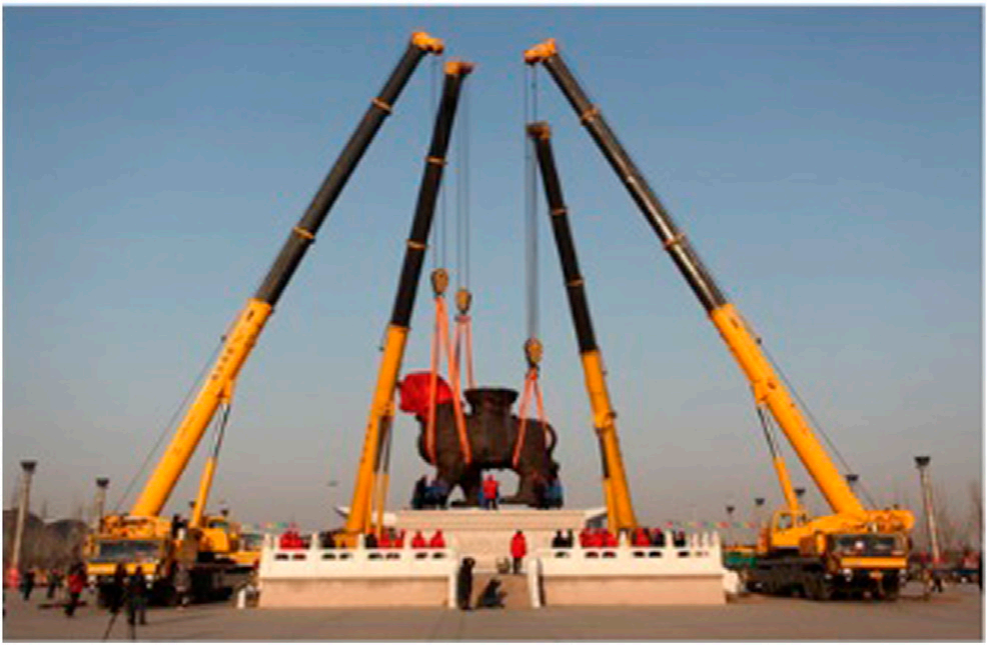
Examples of CDPM systems in use. **(A)** The SkyCam in use at the Washington Huskies Stadium. Image reprinted from Brown (2019). **(B)** A cooperative crane system being used to move an heavy object.

The need for ever increasing load capacities and workspaces is motivating further research on CDPMs which has led to implementation in interesting and challenging industrial and research scenarios. Perhaps the most recognisable application is the SkyCam (Brown, 2019), a camera mount system used in large sports venues and stadiums for live broadcasting (Figure 1A). The system consists of four motorised reels fixed to the corners of the venue that retract or extend the four cables attached to the camera. This allows for three-dimensional control with camera translation speeds of up to 44.8 km/h (Qian et al., 2018) whilst maintaining constant orientation. More recent developments have focused on industrial applications, such as cooperative cable driven crane systems (Qian et al., 2018) (Figure 1), which utilise the large tensile strength of the cable actuators to move heavy payloads. In academia, research at the National Institute of Standards and Technology (NIST) has led to development of the NIST Robocrane, a novel three cable system that has seen many uses including shipping container management on large vessels, load stabilization during transport, and even as a potential modification to lunar rovers for exploration of the moon (NIST, 1994).

### Control Systems for CDPM

As mentioned, replacing rigid links with cables leads to challenges that complicate the design of control systems for CDPMs. Perhaps the most commonly implemented control method is PID control. Khosravi and Taghirad proposed a robust PID controller for a CDPM that controlled the length of each cable, with a corrective term to account for cable elasticity (Khosravi and Taghirad, 2016). The generated controller could stabilize the end effector and showed good orientation control, although desired positional control was not achieved and displayed erratic behaviour. Taking a different approach, Alp and Agrawal proposed a nested closed loop controller based on Lyapunov design and feedback linearization that would output the desired tension in each cable for a given end effector position and orientation (Alp and Agrawal, 2002). This control design allowed for adept positional control with a fast response time and minimal error, but the end effector failed to maintain accurate orientational control. In addition, the controller was complex in design and was hindered by large cable friction during operation. Both of the PID control methods discussed utilised indirect sensing, suffering from a need to estimate the end effector Cartesian pose (position and orientation) from complete knowledge of the inverse kinematics of the cable system, which is highly complex and missing in parts (e.g., Alp and Agrawal did not consider the cable friction in the kinematic model). Newer approaches now consider visual servoing techniques, utilizing computer vision to identify the end effector pose. This simplifies the kinematic model by removing the need to model complex dynamics and instead using simple closed loop feedback techniques to minimize end effector pose error. Dallej et al. reviewed current visual servoing techniques and developed and proposed a vision based PID control system for a ReelAx8 CDPM that was simpler to design and showed good results when assessing the pose errors over time (Dallej et al., 2011).

Previous research was performed on the specific cable rig used in this project by Hong (2019), who was able to design a real time auto tuning PID controller for control of end effector position within the workspace. Here, a Simulink PID auto tuner model was implemented to tune and return optimal gain values for four motors simultaneously in real time. His research showed that a controller was able to able to control the speed the motors attached to each cable with good rise and settling times and minimal steady state error when tested on hardware.

### Ball Balancing Skill Acquisition

The problem of balancing a ball on a plate is an extension of the 2D traditional nonlinear ball on beam balancing problem that is often used as a benchmark in control design theory (Kostamo et al., 2005). The task consists of providing rotational actuation to a beam, where the ball is only free to travel in one axis. For a plate system the ball is free to travel in two axes. With both systems, the goal is to move the ball to a specific location and then maintain its position. As proven by derivation in Awtar et al. (2002) and further documented by Ali and Aphiratsakun (2015), the ball on plate system can be viewed as two independent ball on beam systems provided the plate has mass symmetry about its x-z and y-z axis. As such, both ball on plate and ball on beam systems and control schemes are discussed in this section (Supplementary Figure S1).

#### Classical Control Methods

Multiple attempts have been made to implement PID controllers on both balancing systems with varying degrees of success (Ali and Aphiratsakun, 2015) implemented a basic PID controller onto a ball-plate system that balanced a ball on the center of a plate from a random initial location, and then attempted to recover positional control of the ball after an external disturbance to the plate. The controller performed acceptably and was able to balance the ball in reasonable time for both cases, however the response was extremely oscillatory and took over 30 s to recover positional control from the disturbance. This is likely due to the controller design taking a model free approach and instead tuning the PID controller parameters on the hardware.

Taking a slightly different approach (Shih et al., 2017) developed an embedded PID/PD controller for a ball-beam system. The control structure consisted of a PD controller to choose the desired platform response and then a series of individual PID controllers on each motor to realize the idealized platform response by actuation of the motors. The ball-beam PD controller was tuned on a model of the ball dynamics that was estimated by collecting data on the positional response of the ball to varying inputs. The controller was then tested by placing the ball at one end and having the controller attempt to balance the ball at various locations. It was seen that whilst the controller performed worse when the desired ball location is further from the start point, generally the controller performed well and was able to balance the ball in less than 10 s for all scenarios. The controller did however show consistent initial overshoot in the range 10–20%, indicating the potential for improvement to the control system.

Other attempts have also been made using conventional control theory. In Ryu and Oh (2011), they discuss how estimation of the ball velocity is often a large source of error in ball-beam control systems as it is often estimated as the derivative of the measured ball position. He proposes a state space Linear Quadratic Regulator (LQR) controller as a potential solution to these problems and as a general improvement over standard PID control. The designed controller utilised state estimation of the ball position and velocity to optimize the feedback control system and was then tested by disturbing the balanced system and viewing the ball state reaction. It could be seen that the state estimation for the velocity of the ball was significantly less noisy that than when estimated *via* differentiation. This allowed for a much quicker response, with the ball returning to its balanced position in less than 5 s each time. The results do show small amounts of constant oscillation of the ball position around the set point, but the author suggests this is likely due to friction on the system that has not been modeled.

By studying the above literature, it was discovered that the ball position, its velocity as well as the position of the plate system were crucial when building the above mentioned controllers for balancing the ball. This information served as a bootstrap in defining the reward functions for our reinforcement learning approach.

#### Intelligent Methods

More modern approaches to controller design have focused on producing an “intelligent’” controller that is better suited to the unstable nonlinear system. Rahmat et al. (2010) designed a neural network-based controller and then compared its performance to both PID and LQR controllers on a ball-beam system. The approach consisted of designing a neural network to model the ball dynamics that took inputs of the current ball and beam states and output the future ball states. The model was trained *via* backpropagation. This had the benefit of a model free approach where the dynamics of the ball did not have to be derived, which can often be difficult to quantify. A separate neural network was also designed to control the actuation of the beam orientation based upon the expected output of the model network that was trained using the quasi-Newton backpropagation optimization method. The PID, LQR, and neural network controllers were then all tested and compared. It was seen that whilst all three controllers could successfully balance the ball in less than 5 s with minimal steady state error, the PID controller performance was superior to both other controller types, and the LQR controller was able to achieve a faster response while sacrificing some positional overshoot. This suggested that neural network approaches have the potential to be a valid control solution to this problem but require more work before its performance can be superior to conventional methods. Alternatively, Keshmiri et al. (2012) attempted to combine both traditional control strategies with newer intelligent optimization techniques to develop a superior LQR controller that’s parameters were trained using genetic algorithms. As discussed in the paper, genetic algorithms are a class of stochastic search optimization methods based on random number generation, in this case the search algorithm attempts to find the optimal LQR parameters that minimize the error in position of the ball. The genetic LQR controller was then compared to a PID controller trained using the Ziegler-Nichols method and a normal LQR controller that was trained through trial and error. It was seen in testing that the application of the genetic algorithms allowed for a superior controller that responded faster than PID and LQR controllers with a lower steady state error.

At the forefront of current research is the design of end-to-end neural network controllers. Research on the topic is sparse, showing a clear opportunity to develop a novel solution to this traditional problem. In 2013, a publication by Bigharaz et al. (2013) discussed a neural network-based controller for application to a ball-plate system. It received the ball and plate states as inputs as well as motor control signals as outputs. The paper suggested the neural network controller performs almost as well as a generic PID or fuzzy controller, but the research is limited in its testing and does not discuss the method with which the neural network is trained.

When considering training neural networks, there are predominantly two main methods: optimization and machine learning. Examples of optimization methods are discussed previously, however there is minimal research on the application of machine learning. Machine learning methods consist of training the neural network based upon large sets of data related to the system. In the scenario of the ball-plate system this raises an issue as any dataset is unique to the system it is collected from, and this lack of available data leads to reinforcement learning being a promising method for training neural networks for ball-plate systems.

Publications are sparse on implementation of reinforcement learning to ball-beam or ball-plate systems. This sparsity is surprising due to the current popularity of reinforcement learning which has led to it being applied to a plethora of systems, from goal scoring football robots (Asada et al., 1996) to synthetic human speech bots that are indistinguishable from real voices (Arik et al., 2017).

Its potential benefits in CDPMs are obvious and build upon the benefits of visual servoing, by reducing the need to model and understand the kinematics of the system. Instead, a black box type approach can be taken. By simply monitoring the input and related output (and having an understanding of what the desired output is) a controller can be developed through repeated training that can perform the desired task. Of the limited research performed into the topic, some results show promise for its application. For example, Gullapalli et al. used an unsupervised, direct reinforcement learning algorithm to balance a ball on a one Degree of Freedom (DOF) platform (Gullapalli et al., 1994). Here the system reads the ball position from a series of pressure plates and uses it (along with historic data) to estimate the ball velocity. This, along with the current platform orientation and rate of change of orientation is fed into a neural network trained by a reinforcement learning algorithm that outputs the recommended new orientation, with the goal of returning the ball to the center of the platform. After 700 attempts, the controller learns to balance the ball with no further failures and can run for an indefinite amount of time. No comparison is made to alternate control techniques. Here no modeling has been performed on the ball or platform dynamics, allowing for a much simpler design process.

There is a noticeable gap in research into the application of reinforcement learning to control ball-beam and ball-plate systems, which this paper aims to explore. Developments in this topic could result in improved performance of balancing tasks and control of CDPMs which have many applications in the real world, as discussed throughout this section. This research will also aim to encourage future work on the topic that can further build on the developments made.

## Methodology

### System Overview

**FIGURE 2:**
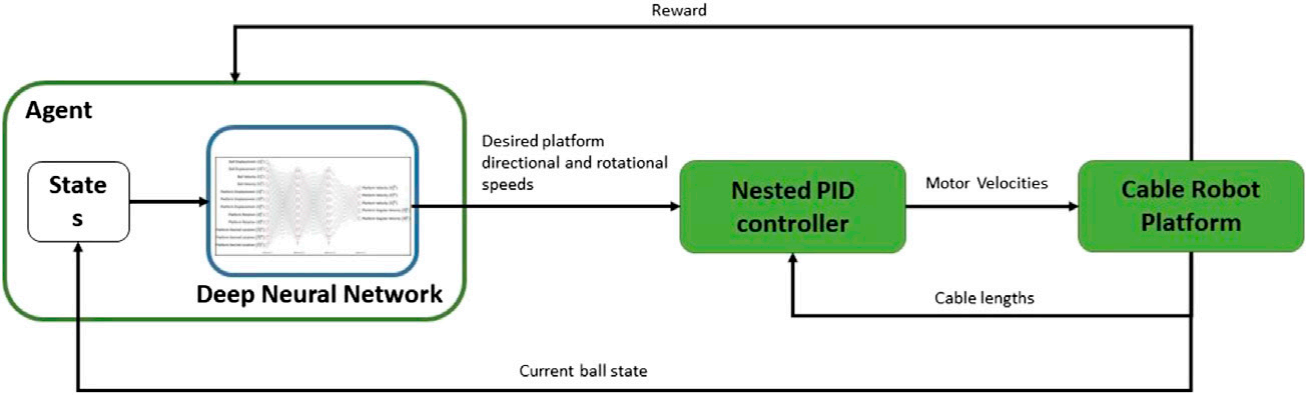
Deep reinforcement learning system diagram for the ball balancing cable robot plant.

The idealized system that meets the aims and objectives set out in *Introduction* section would operate following the system diagram set out in Figure 2. The Agent is the reinforcement learning trained neural network that outputs the desired platform response at the start of each action phase (0.5 s). This is passed to a nested PID controller that realizes the desired platform response for each action phase by controlling the speed of each motor. The nest PID controllers were implemented as a black box with the boxes taking commands from our RL framework. The plant is the physical ball-plate-cable rig.

In Figure 2, the agent receives the platform and ball states. These are used to define reward functions that are then used by the Deep Q learning algorithms to derive a policy that balances the ball (Géron, 2017; Lachevre et al., 2017). In order to balance the ball, the desired direction and rotational speeds are derived by the Q learning algorithm in the form of an optimal policy for use by the Nested PID controller. The Nested PID controller converts these values into motor velocities for use on the cable robot plant. The cable robot plant responds by adapting the length of each cable. *Hardware Design, Ball State Sensing Design, Platform Control Design, and Nested PID Cable Controller Design* sections details the work carried out to realize this system.

### Hardware Design

A preassembled rig was provided at the start of the project that had been used for other CDPM projects. The rig consisted of a fixed frame with a XYZ workspace of 1 m × 1 m × 0.6 m. Fixed above the rig sat a Lego Ev3 Mindstorm microcomputer connected to four Ev3 Large Servo Motors (Supplementary Figure S2). Spools of cable were attached to the motors that extended to the top four corners of the frame. The cables then extended into the workspace where they were connected to another Lego Ev3 Mindstorm. Significant work was carried out on this rig to outfit it for the desired application.

#### Cable Design

As discussed in *Control Systems for CDPM* section one difficult aspect of control for CDPMs is the elasticity of the cables. To avoid the need to model cable elasticity, the first change made was to replace the 0.3 mm diameter string cables with 1 mm nylon cables to significantly reduce cable deformation. The original string material was unknown.

#### Cable Spool Design

The spools used to hold the cables had a diameter of 31.83 mm to achieve 100 mm of cable release per revolution. To achieve a faster system response, the spools were redesigned using CAD software, with roughly twice the diameter of 60 mm to achieve 188.5 mm of cable release per revolution. The designed spools were then 3D printed in polylactide (PLA) plastic (Supplementary Figure S3). In addition, to obtain complete six Degree of Freedom (DoF) control the system needs cable redundancy, as discussed by Enrico Sammarchi. Six DoF is necessary to achieve orientational and positional control of the platform. As such, the number of cables needs to be increased to more than six (since six DoF control is required). Therefore, four additional cables (eight total) were added at the bottom four corners of the workspace that are attached to four additional Lego Large Ev3 motors, making the CDPM a Redundantly Restrained Positioning Mechanism (RRPM) system (Sammarchi, 2019).

#### Platform Design

The current end-effector attached to the cables was an additional Lego Ev3 Mindstorm, which needed to be replaced with a flat plate (Supplemetary Figure S4). The plate was designed in CAD software and laser cut from a 5 mm acrylic sheet. Acrylic was used as it provides a smooth surface and would not flex or crack under usage. The plate was then painted matt black to reduce its reflectiveness to aid ball state extraction *via* image processing by providing a greater contrast with the white ball.

#### Camera Mount Design

Next, to enable identification of ball states, a webcam was obtained with a large field of view (FOV). A large FOV was necessary to reduce the height of the camera mount above the plate and hence reduce its obtrusiveness in the workspace. The mount was designed for the camera in CAD software, and fixes to the underside of the platform. It was designed symmetrically to minimize impact on platform center of gravity and hence bias any motor. The mount fixes to the corners of the plate underside to reduce the likelihood of collision with the ball. The components were then 3D printed in PLA (Supplementary Figures S5, S6).

#### Completed Hardware Model

**FIGURE 3:**
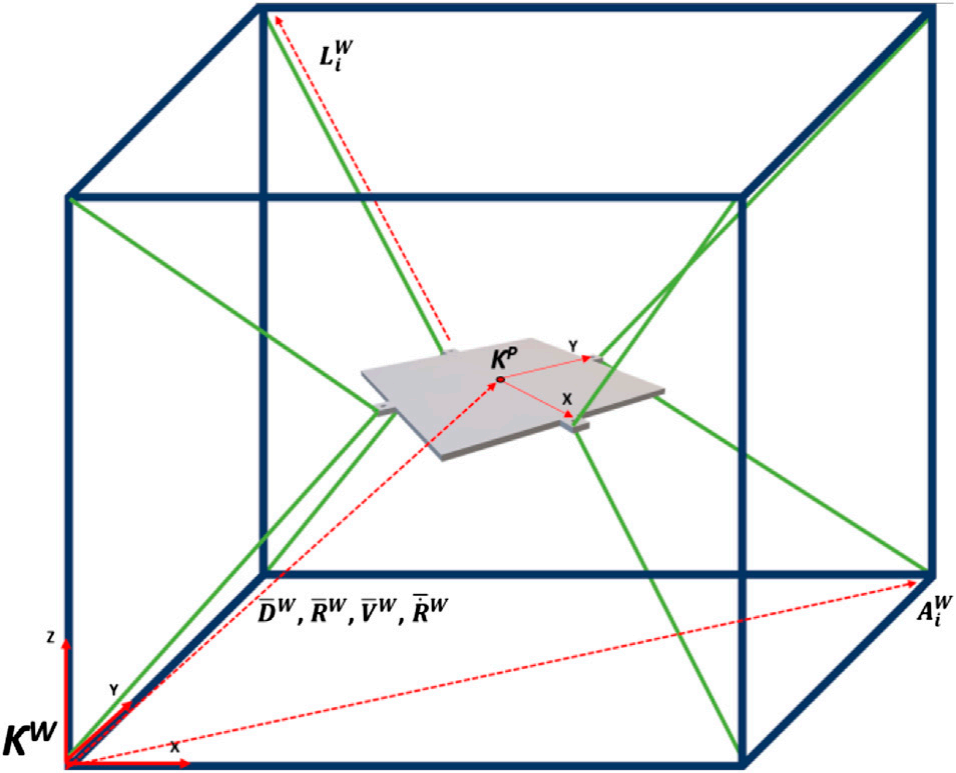
Kinematic model of system with workspace reference frame. Camera mount excluded for ease of viewing.

**FIGURE 4:**
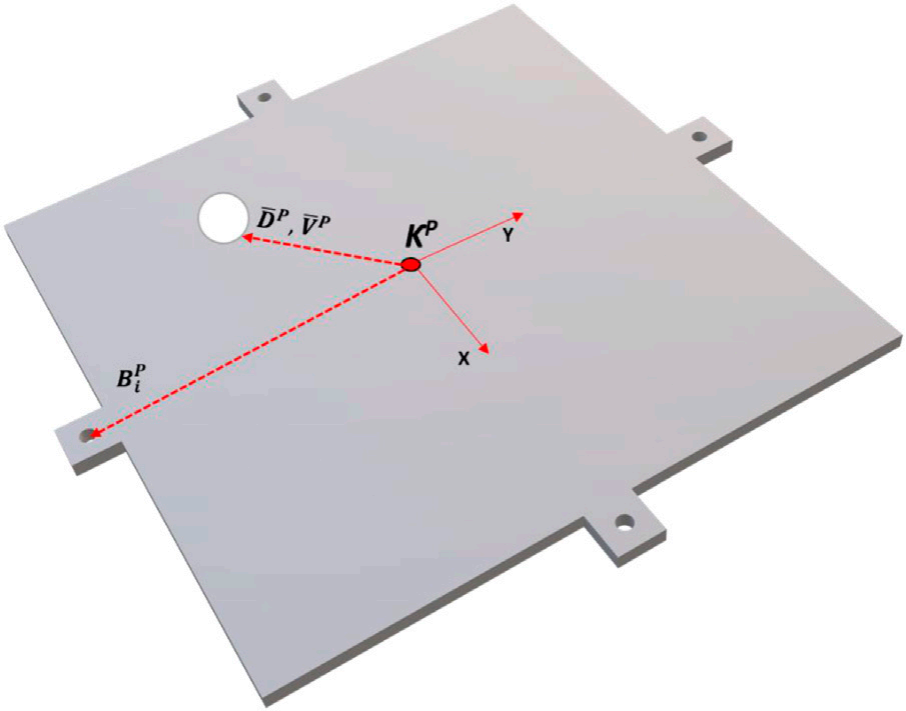
Diagram of ball-plate system with platform reference frame.

The final modified hardware system was assembled. The kinematic model of the system and variable definitions can be seen in Figure 3. Tables 1, 2 show the variable associated with the workspace and the platform references. Figure 4 shows a diagram of the ball-plate system for reference.

**TABLE 1:** Showing workspace reference frame variables.

Platform X displacement: $\left(D_{X}^{W}\right)$	Platform X rotation: $\left(R_{X}^{W}\right)$
Platform Y displacement: $\left(D_{Y}^{W}\right)$	Platform Y rotation: $\left(R_{Y}^{W}\right)$
Platform Z displacement: $\left(D_{Z}^{W}\right)$	
Platform displacement vector:	Platform rotation vector:
${\bar{D}}^{W}={\left[\begin{array}{ccc} D_{X}^{W} & D_{Y}^{W} & D_{Z}^{W} \end{array}\right]}^{T}$	${\bar{R}}^{W}=\left[\begin{array}{ccc} R_{X}^{W} & R_{Y}^{W} & 0{\;}^{T} \end{array}\right]$
Platform X velocity: $\left(V_{X}^{W}\right)$	Platform X angular velocity: $\left({\dot{R}}_{X}^{W}\right)$
Platform Y velocity: $\left(V_{Y}^{W}\right)$	Platform Y angular velocity: $\left({\dot{R}}_{Y}^{W}\right)$
Platform Z velocity: $\left(V_{Z}^{W}\right)$	
Platform velocity vector:	Platform angular velocity vector:
${\bar{V}}^{W}={\left[\begin{array}{ccc} V_{X}^{W} & V_{Y}^{W} & V_{Z}^{W} \end{array}\right]}^{T}$	${\bar{\dot{R}}}^{W}=\left[\begin{array}{ccc} {\dot{R}}_{X}^{W} & {\dot{R}}_{Y}^{W} & 0{\;}^{T} \end{array}\right]$
Cable workspace origins matrix:	
${\bar{A}}^{w}=\left[\begin{array}{c} 0\\ 0\\ 0 \end{array}\;\begin{array}{c} 1000\\ 0\\ 0 \end{array}\;\begin{array}{c} 1000\\ 1000\\ 0 \end{array}\;\begin{array}{c} 0\\ 1000\\ 0 \end{array}\;\begin{array}{c} 0\\ 0\\ 600 \end{array}\;\begin{array}{c} 1000\\ 0\\ 600 \end{array}\;\begin{array}{c} 1000\\ 1000\\ 600 \end{array}\;\begin{array}{c} 0\\ 1000\\ 600 \end{array}\right]$	
$=\left[A_{1}^{W}\ldots \ldots A_{8}^{W}\right]\;$	
Cable lengths matrix:	
${\bar{L}}^{w}=\left[\begin{array}{c} L_{1,X}^{W}\\ L_{1,Y}^{W}\\ L_{1,Z}^{W} \end{array}\;\;\begin{array}{c} L_{2,X}^{W}\\ L_{2,Y}^{W}\\ L_{2,Z}^{W} \end{array}\;\ldots \;\begin{array}{c} L_{8,X}^{W}\\ L_{8,Y}^{W}\\ L_{8,Z}^{W} \end{array}\right]$	
$=\left[L_{1}^{W}\ldots .\;L_{8}^{W}\right]$	

**TABLE 2:** Showing platform reference frame variables.

Ball X displacement: $\left(D_{X}^{P}\right)$	Ball X velocity: $\left(V_{X}^{P}\right)$
Ball Y displacement: $\left(D_{Y}^{P}\right)$	Ball Y velocity: $\left(V_{Y}^{P}\right)$
Ball displacement vector:	Platform velocity vector:
${\bar{D}}^{P}={\left[\begin{array}{ccc} D_{X}^{P} & D_{Y}^{P} & D_{Z}^{P} \end{array}\right]}^{T}$	${\bar{V}}^{P}={\left[\begin{array}{ccc} V_{X}^{P} & V_{Y}^{p} & 0 \end{array}\right]}^{T}$
Plate = 200 mm × 200 mm with 10 mm extensions on each edge for connecting cables. Each connection point is 110 mm from the plate center and is rotated 45° from workspace reference frame. Two cables are connected to each connection point	
Cable plate connection points:	
${\bar{B}}^{P}=\;\left[\begin{array}{c} 0\\ -110\\ 0 \end{array}\;\;\begin{array}{c} 110\\ 0\\ 0 \end{array}\;\;\begin{array}{c} 0\\ 110\\ 0 \end{array}\;\;\begin{array}{c} -110\\ 0\\ 0 \end{array}\;\begin{array}{c} 0\\ -110\\ 0 \end{array}\;\;\begin{array}{c} 110\\ 0\\ 0 \end{array}\;\;\begin{array}{c} 0\\ 110\\ 0 \end{array}\;\;\begin{array}{c} -110\\ 0\\ 0 \end{array}\right]$	
$=\left[B_{1}^{P}\ldots \;B_{4}^{P}\;\;B_{1}^{P}\ldots \;B_{4}^{P}\right]$	

### Ball State Sensing Design

For closed loop feedback control of the ball position, its states need to be identified at the end of every action phase. For state sensing, options such as pressure pads where considered, as used by Gullapalli in his one DOF ball balancing robot (Gullapalli et al., 1994). However, this idea was disregarded due to concerns with compatibility with the Ev3 Brick and the impact it would have on platform design. Instead, a webcam is used alongside an image recognition system.

As Ev3 motor control is being performed in MATLAB, it was decided to also develop the image recognition system using MATLAB and using the Image Processing Toolbox a program was written that takes a still image from the webcam and computes the ball states. The ball position is found by converting the image to a binary image (dependant on pixel luminosity) and examining the binary value of adjacent pixels to identify the “edge” of the ball. Then, using the “regionprops” function the center position of the ball is located. A demonstration of this process can be seen in Supplementary Figure S7. The ball velocity is identified by assessing the change in ball position since the last action phase.

### Platform Control Design

In this section, we design a reinforcement learning trained deep neural network controller that output the desired positional and rotational response to changing ball states at the start of each action phase. This neural network controller is referred to as “the agent.”

#### Neural Network Structure

**FIGURE 5:**
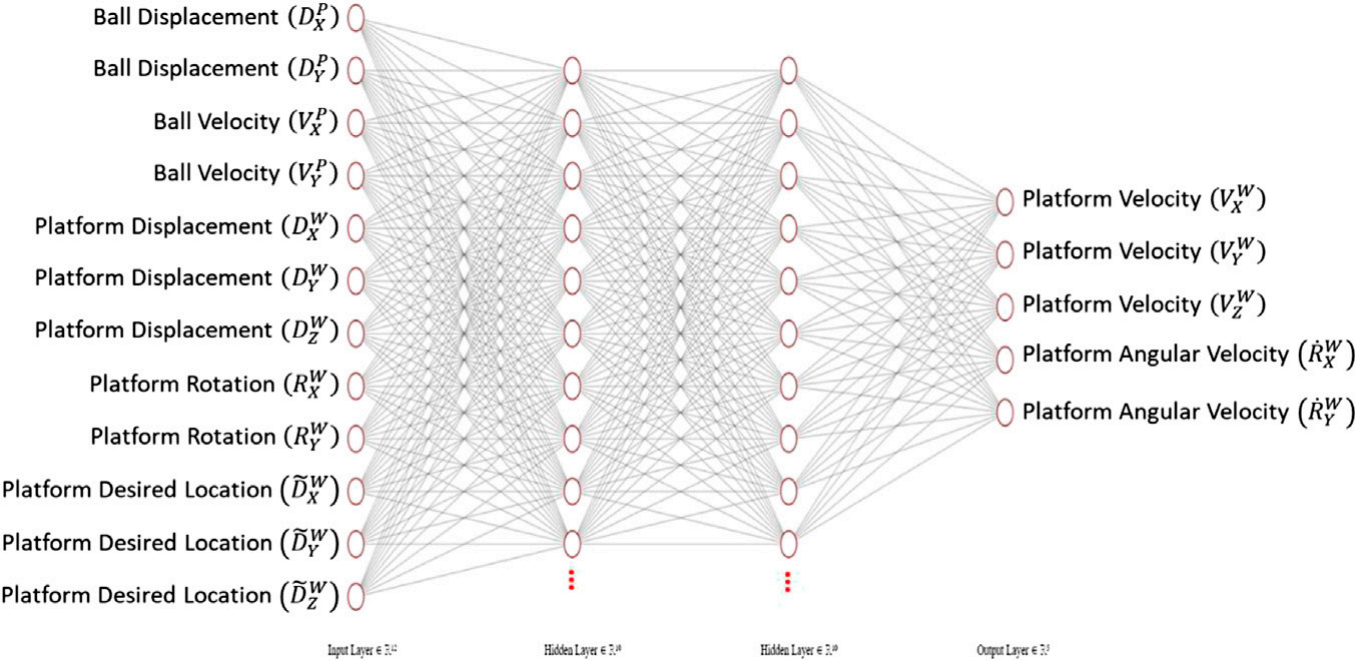
Agent neural network structure for 10 node system.

The structure of the neural network agent is shown in Figure 5. The input layer of the network contains the twelve input states to the system: the ball and plate states and the target end platform location. Two hidden layers are used to make it a deep neural network. Finally, the five node output layer outputs the desired platform response. Note that there is no output function for angular velocity in the *Z* axis as Z orientation is kept constant at the origin, as this offers no benefit in the balancing task. Rectified Linear Units (ReLU) were used as the activation functions. To assess the impact of model complexity on performance, three different model structures were assessed. Each model structure had a varying number of activation functions on each hidden layer: 10, 100, and 500, with an increasing number of activation functions resulting in an increase in model complexity. The weights on each input to each activation function were trained using reinforcement learning.

#### Policy Training Method—Q-Learning and Policy Gradients

As mention in *Ball Balancing Skill Acquisition* section, the process of training a neural network to perform a ball balancing task lends itself naturally to reinforcement learning due to the lack of availability of an appropriate dataset of system responses. It is generally accepted that the two leading approaches for model free reinforcement learning are Q-learning and policy gradients (PG) (DeepMind Technologies, 2014; Lillicrap et al., 2016; Cornell University, 2018).

Q-learning is a reinforcement learning method first introduced in 1989 by Watkins and Dayan (1992) that aims to approximate the Q function of each state-action pair through interacting with the environment. The Q function is the expected sum of future rewards if the policy is followed. Each time the agent interacts with the environment a data point is collected:

$$<s,a,r,s^{\prime }>$$

*s* = the current state of the environment. *a* = the action taken by the agent. *r* = the reward from the environment. *s*′ = the new state of the environment.

The Q function can be iteratively approximated using the Bellman equation through temporal difference learning (Yu, 2017). Once the Q function has been approximated for all state-action pairs, the optimal policy that the agent should take for any given current state can be decided by finding the action that provides the maximum Q function value. It was a deep Q-learning Network (DQN) that famously learned to play a wide range of ATARI games (Mnih et al., 2015) and helped re-popularize the field of reinforcement learning. One of the key drawbacks of Q-Learning is that the Q function needs to be learnt for discrete state-action pair. This means that environments with continuous action spaces require discretization of the states and actions, resulting in a loss of precision of the data (Hodge and Austin, 2012).

Alternatively, PG methods can operate in continuous or discrete action spaces (DeepMind Technologies, 2014) and are becoming the preferred choice for reinforcement learning tasks (Karpathy, 2016). Karpathy suggested that the reason PG methods are becoming favoured is because it is an end-to-end method: there’s an explicit policy and a principled approach that directly optimizes the expected reward (Karpathy, 2016). Instead of estimating the future reward for every state-action pair based upon the data points collected, we estimate the future reward of the policy based on the policy parameters. This then becomes a gradient ascent task where the parameters are tuned to maximize the policy reward (Pseudocode 1). This, alongside the fact that the system operates in a continuous domain (e.g., platform X velocity can be any continuous value in the range of −50 to 50 mm/s) is why a policy gradient method was used for this project.

#### Simulated Environment Design

Whilst this project takes a model free approach to RL agent operation, the ball-plate environment needs to be modeled to allow for training of the agent on simulation. Simulated environment training offers benefits over a purely hardware-based training program in that simulations run drastically faster, can run training exercises simultaneously and require no supervision once initiated.

**FIGURE 6:**
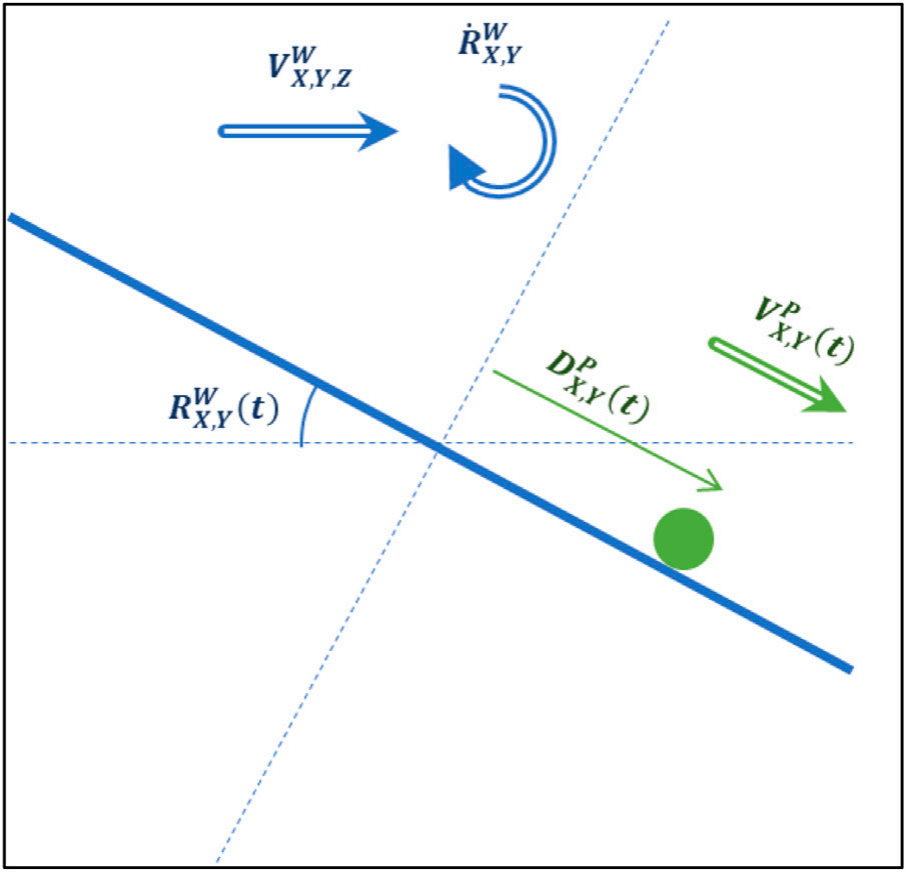
Model of the ball dynamics for the system.

As discussed in *Background and Literature Review* section, the XYZ ball-plate system can be viewed as two independent ball-beam systems operating in the X-Z and Y-Z planes. This ball-beam system is shown in Figure 6. The assumptions which formulate the ball-beam system model are as follows:

1. The ball is always in contact with the plate and does not bounce.
2. There is no slipping motion between the ball and plate
3. Resistive forces on the ball including air resistance and rolling friction are negligible
4. The only force acting on the ball is its weight and the associated reaction force
5. At the start of each action phase, the beam is assumed to undergo an instantaneous change in translational and rotational velocity to the exact desired position. (i.e., the motor response ins assumed to be perfect)
6. The beam maintains its exact desired velocity for the entirety of the action phase
7. Complete knowledge of the ball states is known at all points

Through assumptions 5 and 6, the plate is modeled to have constant velocity and no acceleration, therefore inertial forces from the plate movement on the ball are ignored. Whilst these assumptions are not strictly correct, they are appropriate as the physical plate is limited to small changes in velocities between action phases, and the motors used have high rotational speed so changes in velocity can be assumed close to instantaneous.

From assumption 4, the acceleration of the ball is defined:

$${\dot{V}}_{X,Y}^{P}\left(t\right)=g*\sin\left[R_{X,Y}^{W}\left(t\right)\right]\;for\;t\;\in \;\left\{0,0.5\right\}$$ (1)

Hence the velocity of the ball is found as the Euler integration of Eq. 1

$$V_{X,Y}^{P}\left(t\right)=V_{X,Y}^{P}\left(0\right)+{\dot{V}}_{X,Y}^{P}\left(t\right)\;*t\;for\;t\;\in \;\left\{0,0.5\right\}$$ (2)

And finally, the ball position is found by the Euler integration of Eq. 2:

$$D_{X,Y}^{P}\left(t\right)=D_{X,Y}^{P}\left(0\right)+V_{X,Y}^{P}\left(t\right)\;*t\;\;for\;t\;\in \left\{0,0.5\right\}$$ (3)

From assumptions 6 and 7 the plate dynamical model is seen as:

$$D_{X,Y,Z}^{W}\left(t\right)=D_{X,Y,Z}^{W}\left(0\right)+\;V_{X,Y,Z}^{W}\;*t\;for\;t\;\in \left\{0,0.5\right\}$$ (4)

$$R_{X,Y}^{W}\left(t\right)=R_{X,Y}^{W}\left(0\right)+\;{\dot{R}}_{X,Y}^{W}\;*t\;for\;t\;\in \left\{0,0.5\right\}$$ (5)

Substituting Eq. 5 into Eq. 1 and subsequently Eqs. 2 and 3 gives:

$${\dot{V}}_{X,Y}^{P}\left(t\right)=g*\sin\left[R_{X,Y}^{W}\left(0\right)+\;{\dot{R}}_{X,Y}^{W}\;*t\right]\;for\;t\;\in \left\{0,0.5\right\}$$ (6)

$$V_{X,Y}^{P}\left(t\right)=V_{X,Y}^{P}\left(0\right)+\left\{g*\sin\left[R_{X,Y}^{W}\left(0\right)+\;{\dot{R}}_{X,Y}^{W}\;*t\right]\right\}*t\;for\;t\;\in \left\{0,0.5\right\}$$ (7)

$$D_{X,Y}^{P}\left(t\right)=D_{X,Y}^{P}\left(0\right)+\left[V_{X,Y}^{P}\left(0\right)\;+\left\{g*\sin\left[R_{X,Y}^{W}\left(0\right)+\;{\dot{R}}_{X,Y}^{W}\;*t\right]\right\}*t\right]*t\;for\;t\;\in \left\{0,0.5\right\}$$ (8)

Hence the ball states for any given plate action are given in Eqs. 7 and 8.

This was modeled in a MATLAB environment that was simulated for training.

#### Training Episode Reward

To promote positive actions by the agent, the training process must reward “good” actions and penalise “bad” actions. Toward this, reward functions were defined by taking into consideration the various parameters involved in ensuring the balancing of an unstable load. This included the position of the load, the speed of the load and how the platform positon and orientation affect the load. This resulted in defining three reward components: Ball Position Reward, Ball Speed Reward and Platform Position Reward. For this system, the reward structure was designed to promote balancing of the ball as the platform moves to the target location. As a result, for each action taken in the system, the agent receives a reward consisting of three components:

- **Ball Position Reward:** a reward in the range of (0–1) based upon the Euclidian distance of the ball from the center of the platform, that exponentially decays as the ball moves further from the center
- **Ball Speed Reward:** a reward in the range of (0–1) based on the Euclidian speed of the ball that exponentially decays as the ball speed increases
- **Platform Position Reward:** a reward in the range of (0–1) based on the Euclidian distance of the platform from its desired location that linearly decays

Hence the ball position and speed rewards promote balancing the ball as quickly as possible and the platform position reward promotes transportation of the load as quickly as possible.

The total reward for each action is then the weighted sum of the three components: Action Reward = 6 × Ball Position Reward + 2 × Ball Speed Reward + 2 × Platform Distance RewardAs such each action receives a score between (0–10). If the ball falls off the platform the episode receives a −3,000 reward and the episode is ended.

The reward is weighted to place a larger emphasis on preventing the ball from being dropped as in real world applications dropping the load would cause a larger problem than the speed at which it is delivered. The ball rewards exponentially decay to prioritize keeping the ball away from the platform edge over balancing at the center to prevent episode failure.

#### Agent Training

**FIGURE 7:**
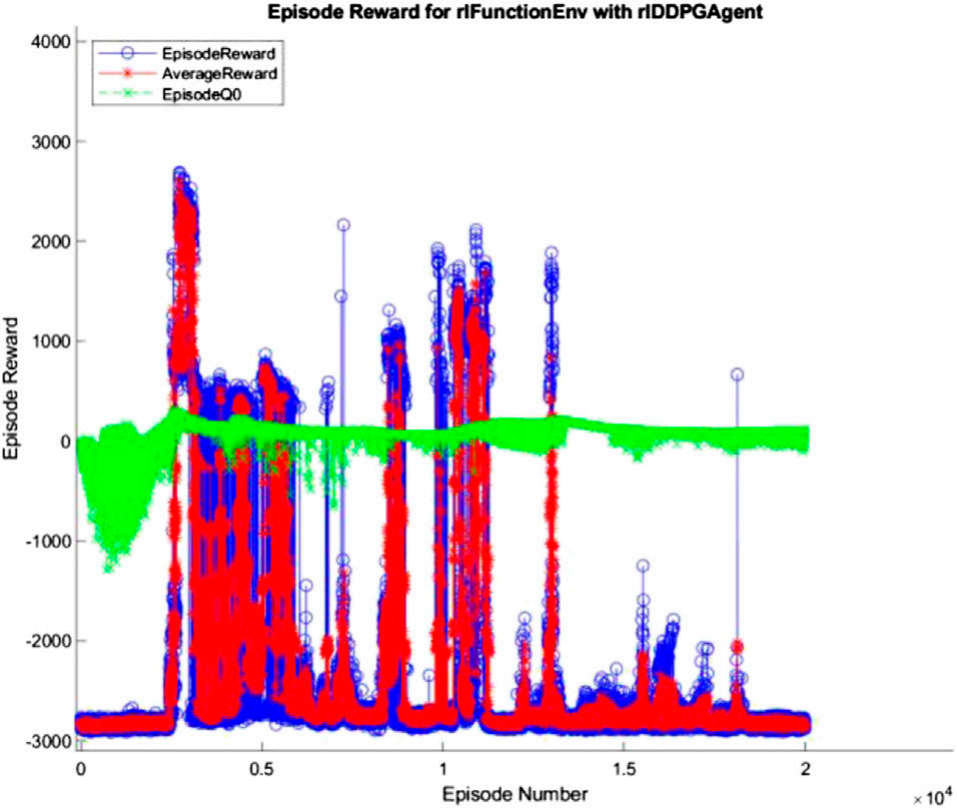
10 node network training progress.

**FIGURE 8:**
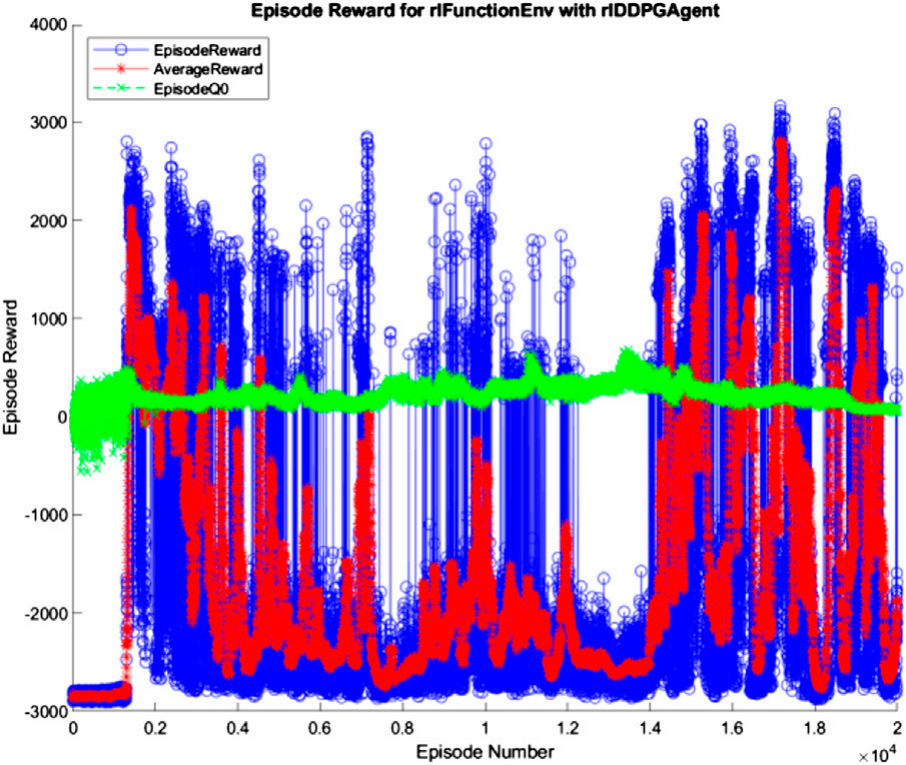
100 node network training progress.

**FIGURE 9:**
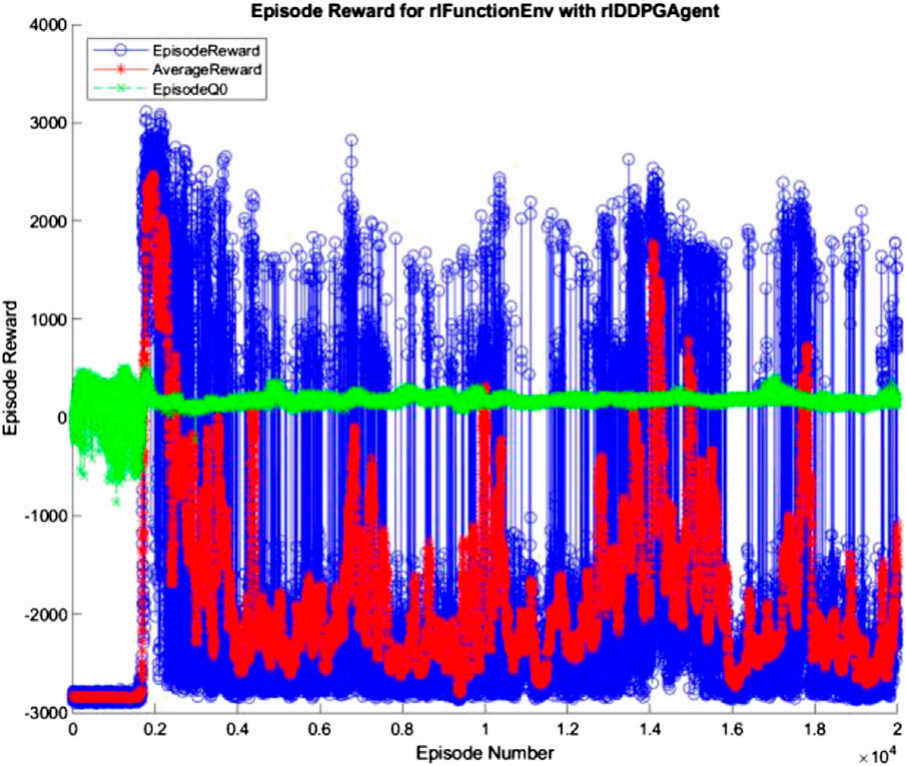
500 node network training progress.

The 10, 100, and 500 node networks were then trained over 20,000 simulated episodes each, with each episode attempting to balance the ball and transport it for 3 min. To accelerate training, four pool parallel processing was used along with GPU acceleration where appropriate. The training progress of each network is shown in Figures 7–9 (The “AverageReward” is a 50-episode rolling average). As each action phase lasts 0.5 s, there are 360 action phases per episode, with a max reward of 3,600 per episode. During training, any agent with an episode reward greater than 2,000 was saved.

All three networks structures can be seen to be erratic between episodes, often identifying strategies that increased episode reward before altering the policy that results in a decrease in performance. The erratic nature of the training suggests the agent struggles to encapsulate the complexity of the system in its policy, with the randomly changing initial conditions of the episode being sufficient variation to cause failure of the controller. Whilst the 100 and 500 node networks also have periodic peaks in performance, the 10 node networks show larger periods between peaks. This suggests increasing model complexity results in an increase in frequency of optimal policy discovery. Overall, all three structures show an inability to consistently converge given an infinite number of training episodes.

### Nested PID Cable Controller Design

As described in the system overview (*System Overview* section), every 0.5 s the agent will output a new desired platform response. This response is actuated through control of the eight motors connected to each of the eight cables, therefore the desired platform response needs to be converted into a desired motor speed.

The first step was to calculate the cable states from the platform states; therefore, the inverse kinematics of the platform were calculated. A reminder of the kinematic model of the system can be seen in Figure 3. It can be seen from literature (Gallardo-Alvarado, 2016; Sammarchi, 2019) that the inverse kinematics for an over defined CDPM is defined as:

$${\bar{L}}^{w}=\;{\bar{A}}^{w}\;-\;{\bar{D}}^{W}\;-\;{\bar{R}}^{W}{\bar{B}}^{P}$$ (9)

Where ${\bar{L}}^{w}$ is a 3 × 8 array of vector lengths of each cable (each row is X/Y/Z and each column is a specific cable). The desired length of each cable can then be calculated as the Euclidian distance:

$$\left|L_{i}^{W}\right|=\;\sqrt{{\left(L_{i,X}^{W}\right)}^{2}+{\left(L_{i,Y}^{W}\right)}^{2}+{\left(L_{i,Z}^{W}\right)}^{2}}$$ (10)

To convert between desired platform and motor response the desired platform location at the start and end of each action phase is calculated and Eqs. 9 and 10 are used to identify the desired initial and final cable lengths. From this the desired motor speed is calculated as:

$$\omega =\frac{L_{final}^{w}-\;L_{initial}^{w}}{0.5\;*\;r}$$ (11)

Where r denotes the radius of the cable spool.

The system was initially designed to utilise the Ev3 inbuilt Tacho feedback that Lego claims performs closed loop control of the motor speed to ensure optimal performance. However, previous research by Hong (2019) on the system revealed consistent steady state error with a poor transient performance. Instead Wei designed and tested a real-time PID controller for motor speed control. This controller was utilized in this project and extended to all eight motors.

## Implementation, Testing and Results

### Image Recognition System Testing

The image recognition system was implemented on the system once the hardware had been constructed. The image recognition system was tested and it was seen that light reflected on the plate resulting in low accuracy for ball position identification. The camera image brightness was reduced, and contrast was increased to mitigate this. In addition, the required gray level for conversion to binary image was increased until the system worked ideally, and was able to identify the ball position in 10 different images.

It is notable that changing the location of the rig would likely result in readjustment of the above parameters which limits the applicability of the system.

### Platform Response Controller Testing

For each network structure, all saved agents were assessed over 500 simulated episodes to identify the agent with the highest average reward for each network structure.

Each simulation started by placing the platform at the center of the workspace with no deviation in orientation. The ball was then placed on the platform at a random position with no initial velocity. The target platform location was randomized within the workspace. The simulation lasted 3 min, or until the ball leaves the platform.

The best performing agent for each network structure was then simulated over a further 10,000 episodes to analyze their performance. The rewards of this testing can be seen in Table 3.

**TABLE 3:** Results of agent training and best episode testing.

Network hidden layer node count	10	100	500
Number of agents with reward > 2,000	31	877	604
Training time	15 h 0 min	66 h 29 min	63 h 55 min^a^
Best performing agent average reward over 10,000 tests	2,681.1	2,812.6	2,670.3
Number of tests where the best performing agent dropped the ball	0	0	56

^a^ Five-hundred node network trained utilizing four pool parallel processing and GPU acceleration for accelerated training.

From these results it can be seen that the 100-node structure achieved a higher average reward than both other network structures. This indicates a superior performance and ability to balance the ball while moving to the desired location. The 10 node and 500 node networks achieved similar average rewards, but the 500-node network failed to balance the ball for the duration of the episode 0.56% of the time. Whilst this is a relatively small failure chance, the consequence of a dropped payload is significant when utilized in industry, and can result in increased costs and risk to personal safety. As such, the 10-node network is superior to the 500-node network. To help understand the actions of each network structure and the systems response the best and worst performing episodes of each structure were further analyzed.

#### 10-Node Agent

**FIGURE 10:**
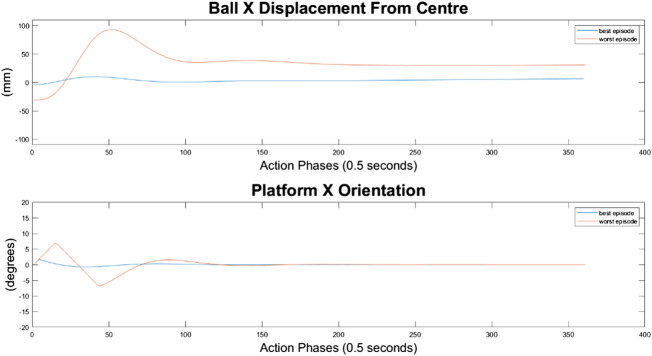
Ball response and change in platform orientation in the *x* axis for the 10 node network.

**FIGURE 11:**
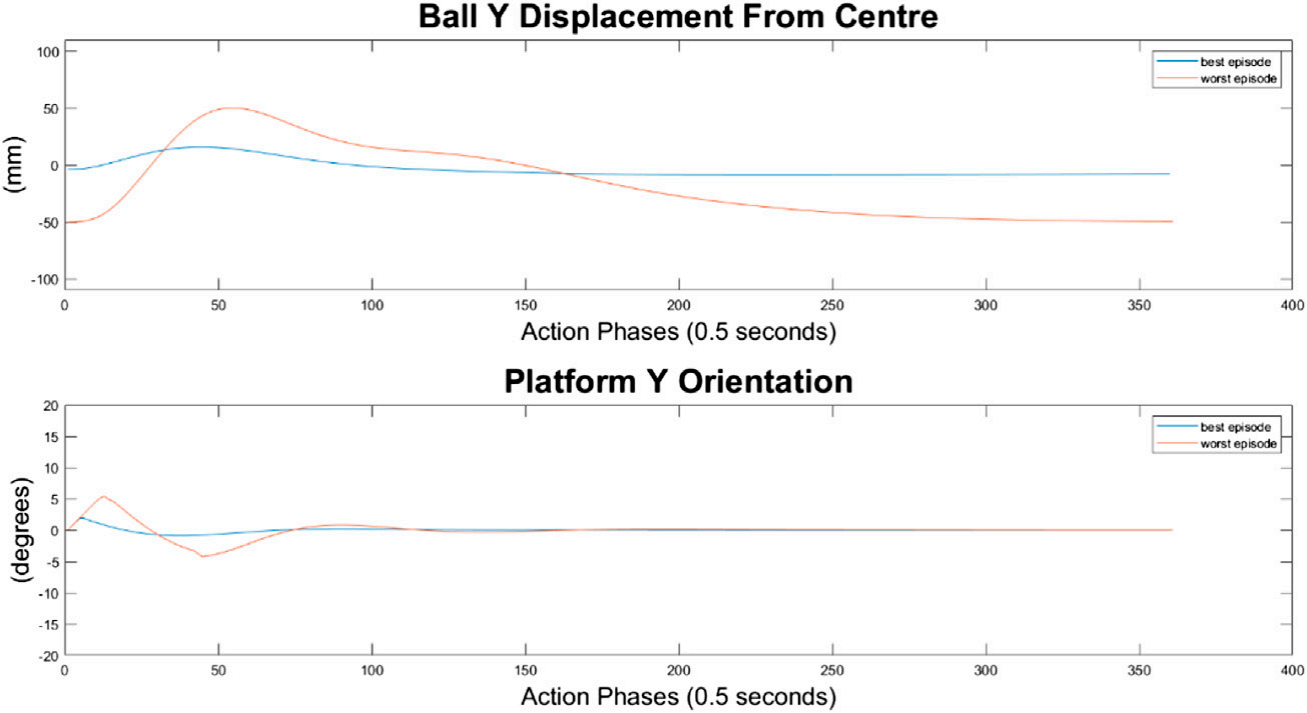
Ball response and change in platform orientation in the *y* axis for the 10 node network.

**FIGURE 12:**
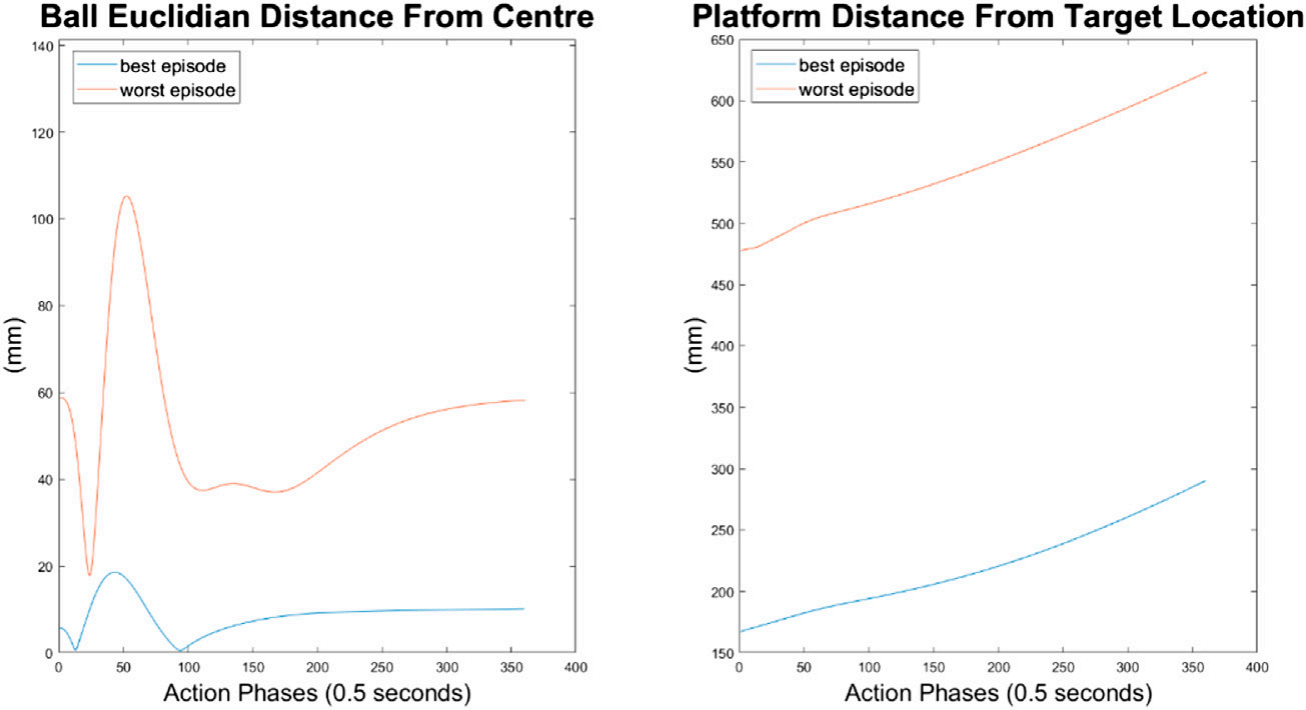
Ball and platform Euclidian distance response for the 10 node network.

As can be seen in Figures 10–12, the 10-node network is capable of balancing the ball, however there is a constant steady state error from the origin, which increases with the balls starting displacement. This error is likely caused by the exponential nature of the reward prescribed based on the balls distance from the origin, described in *Training Episode Reward* section. This in itself is not necessarily a problem, as the ball is balanced somewhere on the platform for the duration of the journey. However, the controller is extremely slow in its ability to reach a steady state ball position and is damped and oscillatory. This is a significant problem as the controller would struggle to respond quickly to any disturbances or knocks it receives during operation. The most notable problem with the controller is that it actively moves the platform away from the target location. As such the controller fails to achieve its key goal of balancing the load as it is transported from one location to another.

#### 100-Node Agent

**FIGURE 13:**
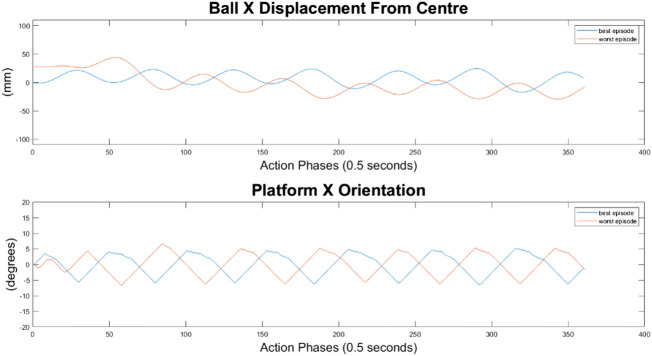
Ball response and change in platform orientation in the *x* axis for the 100 node network.

**FIGURE 14:**
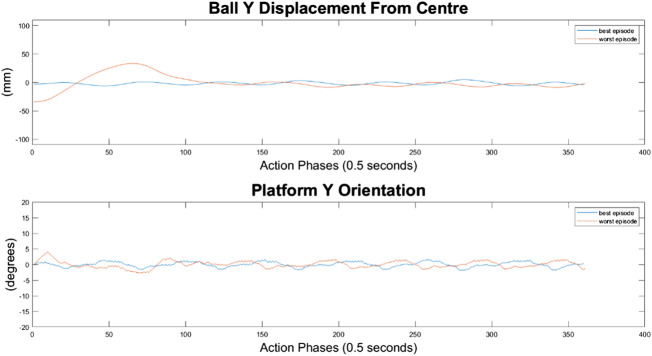
Ball response and change in platform orientation in the *y* axis for the 100 node network.

**FIGURE 15:**
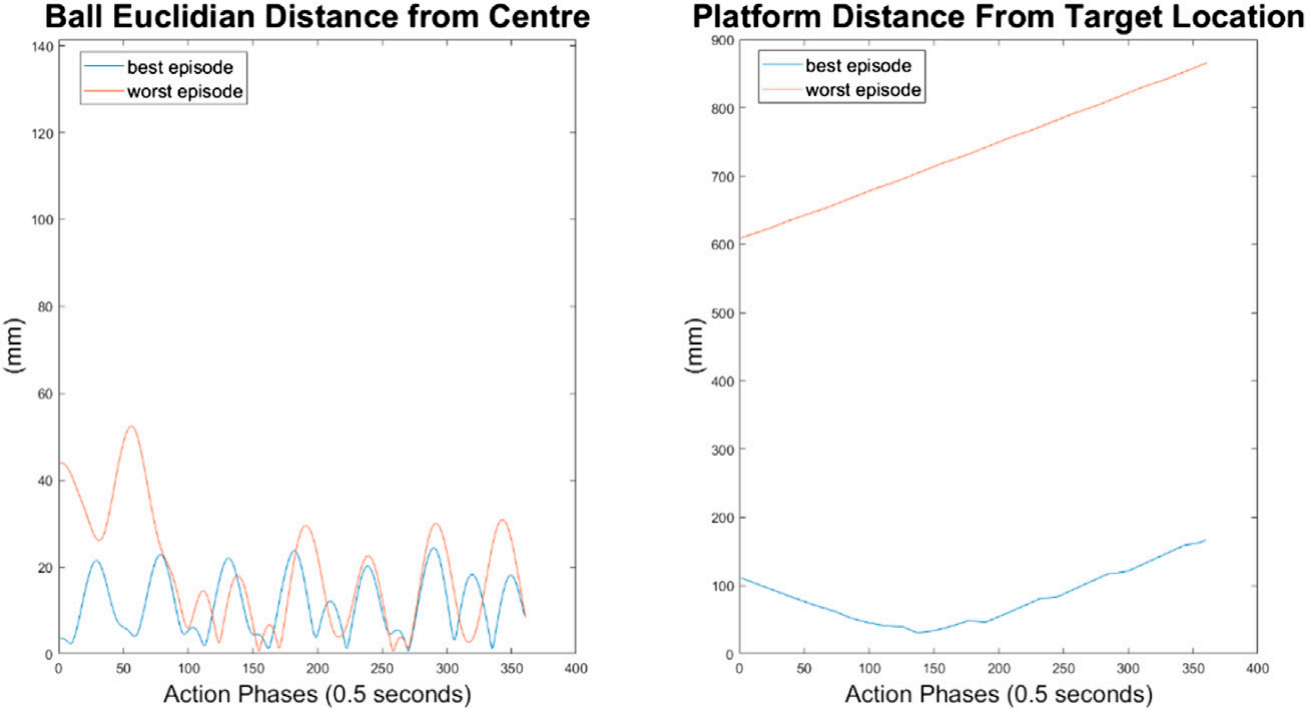
Ball and platform Euclidian distance response for the 100 node network.

It can immediately be seen in Figures 13–15 that the ball response to the 100-node network is much more oscillatory when compared to the 10-node network. Here, the ball fails to reach a stable position and instead continuously oscillates around the origin. Interestingly, the network can control the Y axis ball position better than the X axis position, with smaller oscillations and a damped response to larger displacements. This highlights a flaw in the design choice to have a single complex network to control x-z and y-z states codependently instead of two identical simpler networks controlling x-z and y-z states independently.

The higher frequency of platform oscillation suggests a system more capable of adapting to disturbances, however it also suggests a more unstable controller that may fail under more fringe circumstances. When considering the system’s ability to move the platform between two locations, the 100-node network controller does show slight improvements as can be seen by the best episode showing an initial reduction in platform distance. However over time the platform moves further away from the target, so still fails to transfer the load from one location to another.

#### 500-Node Agent

**FIGURE 16:**
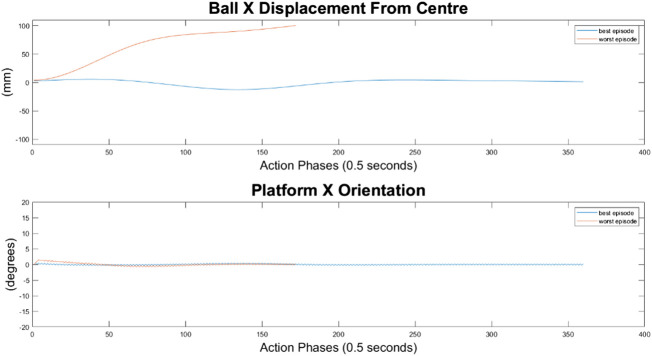
Ball response and change in platform orientation in the *x* axis for the 500 node network.

**FIGURE 17:**
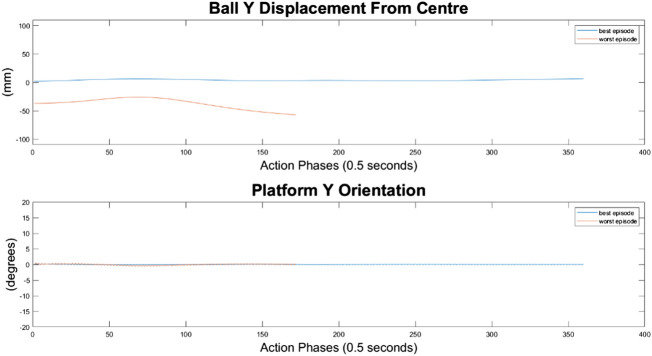
Ball response and change in platform orientation in the *y* axis for the 500 node network.

**FIGURE 18:**
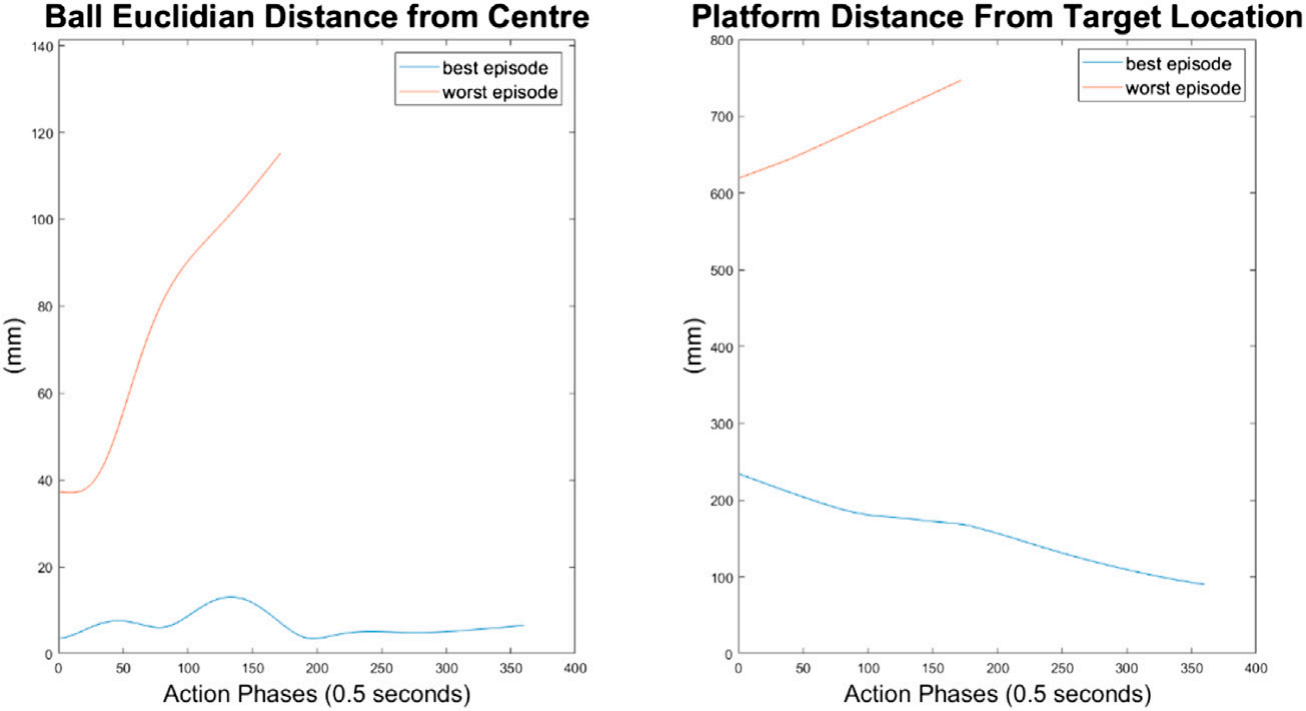
Ball and platform Euclidian distance response for the 500 node network.

For the 500-node network, the worst response recorded was when the ball fell off the platform from the positive X axis edge. The ball states are noticeably less oscillatory when compared to the 100-node network, however the platform response is extremely responsive with high frequency, low amplitude oscillations dominating the orientation response. This platform response results in a slow responding low frequency oscillatory response from the ball as it does not have time between samples to achieve any noticeable velocity. Disregarding the failure cases, the 500-node networks appears to achieve a relatively minimal steady state error in ball position, as can be seen in Figures 16–18. In addition, it shows promise when analyzing its ability to move the platform to the desired location, but the platform’s failure to always balance the load suggests it is an inferior controller to the 10 and 100-node networks.

## Discussion

The results of the performed testing suggest that whilst the reinforcement learning controller showed promise for balancing a load on a CDPM, in its current state it is not an improvement on the state of the art. The controller failed to outperform in key performance metrics including rise time and steady state error.

Alex Irpan, a software engineer on the robotics team at Google Brain, explained how important having a well-designed reward criteria is for RL (Irpan, 2018), describing how it has a tendency to overfit to your reward. In post-review of this project, one area of fault may be the reward structure. Since RL aims to achieve the highest reward possible, the reward function should capture the exact desired outcome. This was not the case in this project, as too high a priority was placed upon the system’s ability to balance the ball instead of transporting the load. In addition, the velocity-based reward was unnecessary and counterproductive as it discouraged the system from attempting to move the ball closer to the center of the platform quickly. With the overfitting nature of RL to the reward in mind, a simpler reward function may improve training, where the velocity reward is removed, and the ball and plate displacement rewards are weighted equally. The complexity of the reward structure can be seen in the training progress described in Figures 7–9 in *Hardware Design* section, where the reward was erratic and unsustainable, suggesting the existence of multiple local optima for the problem.

This project was a great exercise in the impact of model complexity on performance. It could be seen how less complex models produced less complex actions (i.e., smaller changes in output), whereas more complex models were erratic and responsive, sometime to their own detriment. Nevertheless, overly simple models also lead to underfitted controllers whose response are not adequate. Whilst no testing was performed on hardware, we suspect that the more complex controllers would see the greatest drop in performance from the simulated testing as the varied environment would likely expose instability within the controller. The controller would likely be overfitted to the reward structure and modeled environment, which was not absolutely true to the real world. On the flipside, the simpler agent structures struggled to wholly capture the simulated environment which led to a worse performing system as it was slower to respond.

Agent performance on the actual rig will vary due to assumptions made during environment modeling. As discussed, the model assumed no inertial forces on the ball due to platform displacement. These assumptions where justified in *Platform Control Design* section, and we maintain the belief that these assumptions are valid, however it is worth noting that as the system response increases in magnitude then the assumptions become less valid, limiting the applicability of this work.

The design process for this system was laboured and highlighted how implementation of intelligent systems on custom build hardware can often be the most time-consuming task in prototype-based research projects. The use of CAD and 3D printing is highlighted throughout this project and made a noticeable difference on our ability to complete the design and build tasks in a reasonable timeframe.

## Conclusion

In the future, structures might need to be constructed rapidly in response to emergencies such as Pandemics. In order to reduce infection rates, worker’s exposure to dangerous environments and build rapidly, robotic devices such as Cable Driven Parallel Manipulators could be deployed. These devices would need to deal with a variety of challenges including transporting highly unstable loads. In this work, we have designed and built a CDPM controlled by a neural network reference controller with a nested PID controller, to balance a load on a platform as it is transferred from one location to another. The neural network controller was a two-layer deep network with varying model complexity that was trained using a Deep Deterministic Policy Gradient (DDPG) reinforcement learning algorithm. The neural network controller was trained and assessed over a simulated environment. The system utilized image recognition techniques to identify the states of the loads for feedback and platform states were identified by calculating the cable inverse kinematics.

The image recognition system was able to accurately identify the ball states, however the system was vulnerable to calibration problems due to changing lighting conditions. This may affect the applicability of the system, but would not cause an issue if used in environment with a consistent light source, such as an enclosed warehouse.

Assessments of network complexity showed how precise choosing of model parameters is needed to ensure adequate capturing of the real system, and how overfitting and underfitting are likely when training is performed entirely on simulation. In the future, we plan to investigate how neural network complexity contributes to controller efficiency.

In this work, it was seen that the neural network controller was capable of balancing a load, however the performance did not show any significant improvements on the state of the art. In addition, the controller failed to transport the load, which was a key requirement. The attempt showed how there was still promise for the utilization of RL in ball balancing tasks but highlighted how design of RL systems is difficult and often leads to ineffective solutions, especially for complex systems.

Future work on the system should firstly prioritize testing and implementation of the system on the real rig. Additional work may wish to look at redesigning the reward structure and network for simpler performance. Furthermore, identification of the forward kinematics (i.e., calculating the platform pose from the cable lengths) could lead to the removal of the nested PID controller and introduce an end-to-end neural network controller that would output direct motor control signals. Also, investigating and comparing the combination of RL with other heuristics in the policy search, such as the bacterial foraging algorithm (Oyekan and Hu, 2010; Oyekan et al. 2013), will be carried out in the future.

## Data Availability

The raw data supporting the conclusions of this article will be made available by the authors, without undue reservation.
