# Supplementary material for: Applying Deep Reinforcement Learning to Cable Driven Parallel Robots for Balancing Unstable Loads: A Ball Case Study
Source: Front Robot AI. 2021 Feb 22;7:611203. doi: 10.3389/frobt.2020.611203 (PMC7938313; doi:10.3389/frobt.2020.611203)
Supplement: Supplementary file 1 [file table1.docx]

| **Table 1. Showing workspace reference frame variables.** | |
| --- | --- |
| Platform X Displacement: $\left( D_{X}^{W} \right)$  Platform Y Displacement: $\left( D_{Y}^{W} \right)$  Platform Z Displacement: $\left( D_{Z}^{W} \right)$  Platform Displacement Vector:  $\bar{D}^{W}=[\begin{matrix} D_{X}^{W} & D_{Y}^{W} & D_{Z}^{W} \end{matrix}]^{T}$  Platform X Velocity: $\left( V_{X}^{W} \right)$  Platform Y Velocity: $\left( V_{Y}^{W} \right)$  Platform Z Velocity: $\left( V_{Z}^{W} \right)$  Platform Velocity Vector:  $\bar{V}^{W}=[\begin{matrix} V_{X}^{W} & V_{Y}^{W} & V_{Z}^{W} \end{matrix}]^{T}$ | Platform X Rotation: $\left( R_{X}^{W} \right)$  Platform Y Rotation: $\left( R_{Y}^{W} \right)$  Platform Rotation Vector:  $\bar{R}^{W}=[\begin{matrix} R_{X}^{W} & R_{Y}^{W} & 0 ]^{T} \end{matrix}$  Platform X Angular Velocity: $\left( \dot{R}_{X}^{W} \right)$  Platform Y Angular Velocity: $\left( \dot{R}_{Y}^{W} \right)$  Platform Angular Velocity Vector:  ${\bar{\dot{R}}}^{W}=[\begin{matrix} \dot{R}_{X}^{W} & \dot{R}_{Y}^{W} & 0 ]^{T} \end{matrix}$ |
| Cable Workspace Origins Matrix:  $\bar{A}^{w}=\left[ \begin{matrix} 0 \\ 0 \\ 0 \end{matrix} \begin{matrix} 1000 \\ 0 \\ 0 \end{matrix} \begin{matrix} 1000 \\ 1000 \\ 0 \end{matrix} \begin{matrix} 0 \\ 1000 \\ 0 \end{matrix} \begin{matrix} 0 \\ 0 \\ 600 \end{matrix} \begin{matrix} 1000 \\ 0 \\ 600 \end{matrix} \begin{matrix} 1000 \\ 1000 \\ 600 \end{matrix} \begin{matrix} 0 \\ 1000 \\ 600 \end{matrix} \right]$  $=[ A_{1}^{W}\ldots\ldots A_{8}^{W} ]$  Cable Lengths Matrix:  $\bar{L}^{w}=\left[ \begin{matrix} L_{1,X}^{W} \\ L_{1,Y}^{W} \\ L_{1,Z}^{W} \end{matrix} \begin{matrix} L_{2,X}^{W} \\ L_{2,Y}^{W} \\ L_{2,Z}^{W} \end{matrix} \ldots\begin{matrix} L_{8,X}^{W} \\ L_{8,Y}^{W} \\ L_{8,Z}^{W} \end{matrix} \right]$  $=\left[ L_{1}^{W}\ldots. L_{8}^{W} \right]$ | |
