# Supplementary material for: Applying Deep Reinforcement Learning to Cable Driven Parallel Robots for Balancing Unstable Loads: A Ball Case Study
Source: Front Robot AI. 2021 Feb 22;7:611203. doi: 10.3389/frobt.2020.611203 (PMC7938313; doi:10.3389/frobt.2020.611203)
Supplement: Supplementary file 2 [file table2.docx]

| **Table 2. Showing platform reference frame variables** | |
| --- | --- |
| Ball X Displacement: $\left( D_{X}^{P} \right)$  Ball Y Displacement: $\left( D_{Y}^{P} \right)$  Ball Displacement vector:  $\bar{D}^{P}=[\begin{matrix} D_{X}^{P} & D_{Y}^{P} & D_{Z}^{P} \end{matrix}]^{T}$ | Ball X Velocity: $\left( V_{X}^{P} \right)$  Ball Y Velocity: $\left( V_{Y}^{P} \right)$  Platform Velocity Vector:  $\bar{V}^{P}=[\begin{matrix} V_{X}^{P} & V_{Y}^{p} & 0 \end{matrix}]^{T}$ |
| Plate = 200mm x 200mm with 10mm extensions on each edge for connecting cables. Each connection point is 110mm from the plate centre and is rotated 45° from workspace reference frame. 2 cables are connected to each connection point  Cable Plate Connection Points:  $\bar{B}^{P}= \left[ \begin{matrix} 0 \\ -110 \\ 0 \end{matrix} \begin{matrix} 110 \\ 0 \\ 0 \end{matrix} \begin{matrix} 0 \\ 110 \\ 0 \end{matrix} \begin{matrix} -110 \\ 0 \\ 0 \end{matrix} \begin{matrix} 0 \\ -110 \\ 0 \end{matrix} \begin{matrix} 110 \\ 0 \\ 0 \end{matrix} \begin{matrix} 0 \\ 110 \\ 0 \end{matrix} \begin{matrix} -110 \\ 0 \\ 0 \end{matrix} \right]$  $=[B_{1}^{P}\ldots B_{4}^{P} B_{1}^{P}\ldots B_{4}^{P}]$ | |
