# Supplementary material for: Applying Deep Reinforcement Learning to Cable Driven Parallel Robots for Balancing Unstable Loads: A Ball Case Study
Source: Front Robot AI. 2021 Feb 22;7:611203. doi: 10.3389/frobt.2020.611203 (PMC7938313; doi:10.3389/frobt.2020.611203)
Supplement: Supplementary file 3 [file table3.docx]

Table 3.Results of agent training and best episode testing

**500 node network trained utilising 4 pool parallel processing and GPU acceleration for accelerated training*

| Network Hidden Layer Node Count | 10 | 100 | 500 |
| --- | --- | --- | --- |
| Number of agents with reward > 2000 | 31 | 877 | 604 |
| Training time | 15 hr 0 min | 66 hr 29 min | 63 hr 55 min * |
| Best performing agent average reward over 10,000 tests | 2,681.1 | 2,812.6 | 2,670.3 |
| Number of tests where the best performing agent dropped the ball | 0 | 0 | 56 |
